# Effects of Metabotropic Glutamate Receptor 3 Genotype on Phonetic Mismatch Negativity

**DOI:** 10.1371/journal.pone.0024929

**Published:** 2011-10-11

**Authors:** Yuki Kawakubo, Motomu Suga, Mamoru Tochigi, Masato Yumoto, Kenji Itoh, Tsukasa Sasaki, Yukiko Kano, Kiyoto Kasai

**Affiliations:** 1 Department of Child Neuropsychiatry, University of Tokyo, Tokyo, Japan; 2 Department of Neuropsychiatry, University of Tokyo, Tokyo, Japan; 3 Department of Laboratory Medicine, University of Tokyo, Tokyo, Japan; 4 Department of Cognitive and Speech Sciences, University of Tokyo, Tokyo, Japan; 5 Health Service Center, University of Tokyo, Tokyo, Japan; Chiba University Center for Forensic Mental Health, Japan

## Abstract

**Background:**

The genetic and molecular basis of glutamatergic dysfunction is one key to understand schizophrenia, with the identification of an intermediate phenotype being an essential step. Mismatch negativity (MMN) or its magnetic counterpart, magnetic mismatch field (MMF) is an index of preattentive change detection processes in the auditory cortex and is generated through glutamatergic neurotransmission. We have previously shown that MMN/MMF in response to phoneme change is markedly reduced in schizophrenia. Variations in metabotropic glutamate receptor (GRM3) may be associated with schizophrenia, and has been shown to affect cortical function. Here we investigated the effect of *GRM3* genotypes on phonetic MMF in healthy men.

**Methods:**

MMF in response to phoneme change was recorded using magnetoencephalography in 41 right-handed healthy Japanese men. Based on previous genetic association studies in schizophrenia, 4 candidate SNPs (rs6465084, rs2299225, rs1468412, rs274622) were genotyped.

**Results:**

*GRM3* rs274622 genotype variations significantly predicted MMF strengths (p = 0.009), with C carriers exhibiting significantly larger MMF strengths in both hemispheres compared to the TT subjects.

**Conclusions:**

These results suggest that variations in *GRM3* genotype modulate the auditory cortical response to phoneme change in humans. MMN/MMF, particularly those in response to speech sounds, may be a promising and sensitive intermediate phenotype for clarifying glutamatergic dysfunction in schizophrenia.

## Introduction

Synaptic pathology through glutamatergic dysfunction is a key to understand the pathophysiology of schizophrenia [1]. Functions of susceptibility genes for schizophrenia have converged to synaptic plasticity and glutamatergic neurotransmission [2]. Postmortem studies have shown reduced spine density in prefrontal and auditory cortices in schizophrenia [3], [4].

Consistently observed reduction of mismatch negativity (MMN) event-related potentials or its magnetic counterpart (magnetic mismatch field; MMF), an index of auditory sensory memory with a major generator in the auditory cortex, has supported the hypothesis [5], since the generation of MMN is regulated through the N-methyl-D-aspartate (NMDA) receptor agonistic effect [6]. Thus, MMN/MMF may be a useful intermediate phenotype to assess glutamatergic function in schizophrenia.

MMN/MMF is also elicited by change in speech sounds [7], which has been recognized as an index of language-specific speech-sound traces and learning-induced short-term plasticity. We have shown that phonetic MMN, rather than tonal MMN, exhibited more marked reduction in schizophrenia [8]. We replicated the findings using MMF [9]. Moreover, the reduced MMF strengths were significantly associated with reduced left planum temporale gray matter volume reduction in patients with schizophrenia [10]. These results suggest that MMN/MMF in response to phoneme change may be a promising candidate as an intermediate phenotype of glutamate-related genes.

Metabotropic glutamate receptors, by augmenting the function of, or co-acting with, NMDA receptors, are important in synaptic plasticity [1]. Metabotropic glutamate receptor 3 (*GRM3*) may be associated with schizophrenia phenotype [11]–[14], although controversial [15], [16]. Among studies reporting positive findings, the polymorphisms showing association were totally different. Egan *et al.* [12] observed that the single nucleotide polymorphism (SNP) rs6465084 (hCV11245618) and a related haplotype are associated with the disease in a U.S. population. The same SNP was studied by Norton *et al.* [15], and no significant association was observed. In Asians, Chen *et al.* [13] observed a significant association of the SNP rs2299225 and a related haplotype with schizophrenia in Chinese subjects. Fujii *et al.* [11] found an association between rs1468412 and schizophrenia in their Japanese study. Bishop *et al.* [14] has shown that *GRM3* rs274622 modulated the effect of olanzapine on negative symptoms in Caucasian patients with schizophrenia.

Moreover, *GRM3* genotype modulates prefrontal BOLD signals and N-Acetylaspartate (NAA) levels [12], [17] and mGlu3 protein levels were reduced in the prefrontal cortex in postmortem brains of schizophrenia [18].

Accordingly, we predicted that phonetic MMF would be modulated by variations in *GRM3* in healthy subjects. Our hypothesis stems from the facts: 1) we previously found more marked reduction of phonetic MMN rather than tonal MMN in patients with schizophrenia [8], [9]; 2) phonetic MMN has been recognized as an index of language-specific speech-sound traces and learning-induced short-term plasticity [7]; 3) MMN is thought to be a promising intermediate phenotype for glutamatergic system which is involved in synaptic plasticity [5]. We addressed all of the SNPs that showed significant results in previous studies [11]–[14].

## Methods

### Subjects

Participants were 41 healthy Japanese men (mean age: 28.8+/−5.5 years). All participants were right-handed according to the Edinburgh Inventory and were native Japanese speakers. The average years of education of the subjects were 17.0 [SD = 1.2]; the average IQ was 112.3 [SD = 7.6]. For screening of healthy subjects, SCID non-patient edition (SCID-NP) was used. Exclusion criteria were neurological illness, hearing dysfunction, traumatic brain injury with any known cognitive consequences or loss of consciousness for more than 5 minutes, a history of substance abuse or addiction. The ethical committee of the Faculty of Medicine, University of Tokyo approved this study (No. 784-2 for MEG experiment; No. 639-9 for imaging-genetics project). All subjects gave written informed consent after a complete explanation of the study.

### MEG recording and analysis

MEG recording and analysis was described in detail elsewhere [19]. Briefly, the subjects were presented with sequences of auditory stimuli to both ears, consisting of standard (Japanese vowel /a/ with a 250-msec duration, 80 dB SPL and a rise/fall time of 10 ms; probability = 90%) and deviant (Japanese vowel /o/ with a 250-msec duration, p = 10%) stimuli. The stimulus onset asynchrony was 445±15 msec. Measurements were performed in the early afternoon (from 2 p.m. through 3 p.m.). The subjects were instructed to perform a visual detection task, in order to keep attention away from the auditory stimuli. MEG signals were recorded using VectorView (Elekta Neuromag, Helsinki, Finland), which has 204 first-order planar gradiometers at 102 measuring sites on a helmet-shaped surface that covers the entire scalp. The recorded data were filtered online with a band-pass filter of 0.03–100 Hz, digitalized at a sampling rate of 512 Hz, and averaged online separately for standard and deviant stimuli. The duration of the averaging period was 400 ms, including an 80-msec prestimulus baseline. Trials with EOG movement exceeding 150 µV or MEG exceeding 3,000 fT/cm were excluded the analysis. The number of accepted responses for deviant stimuli was above 100 for all subjects. The averaged data were further filtered offline with a band-pass filter of 1–20 Hz.

The MMF is defined as the difference between the evoked magnetic fields of the standard stimuli and those of the deviant tone. The strength of MMF was indexed by the magnetic counterpart of the global field power (mGFP), which was calculated as the root mean squares of the differences over the 54 channels positioned over the temporal region, separately for each hemisphere [9], [20].

### Genotyping

Genotyping procedures were also described in detail elsewhere [16]. Briefly, genomic DNA was extracted from leukocytes by using the standard phenol-chloroform method. Based on previous literature, we genotyped 4 candidate SNPs (rs6465084, rs2299225, rs1468412, rs274622) of *GRM3*.

### Statistical analysis

The mixed model repeated measures analysis of variance (ANOVA) was performed on the mGFPs of MMF, adopting the genotype as a between-subject factor and the hemisphere as a within-subject factor. For rs6465084, AA subjects (N = 33) and AG subjects (N = 8) were compared; for rs2299225, TT subjects (N = 33) and TG subjects (N = 8) were compared; for rs1468412, AA subjects (N = 25) and AT/TT subjects (N = 16 [AT = 15/TT = 1]) were compared; for rs274622, CC/CT subjects (N = 19 [CC = 2/CT = 17]) and TT subjects (N = 22) were compared. The threshold for statistical significance was set at p<0.0125 based on the Bonferroni correction.

## Results

**Figure 1:**
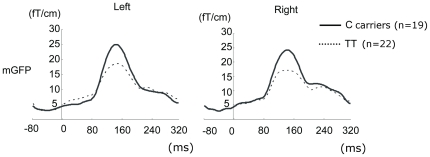
Grand mean mGFP waveforms of MMF. These waveforms were averaged for each GRM3 rs274622 genotype group for each hemisphere (C carriers [N = 19], solid line; TT individuals [N = 22], dashed line).

Figure 1 shows grand mean mGFP waveforms of MMF averaged for each GRM3 rs274622 genotype group for each hemisphere. The repeated measures ANOVA showed a significant main effect of *GRM3* rs274622 genotype (F[1,39] = 7.59, p = 0.009), with no genotype X hemisphere interaction (F[1,39] = 0.036, p = 0.85). The *GRM3* rs274622 C carriers (CC+TC) exhibited significantly larger mGFP values in both hemispheres compared to the TT subjects (post-hoc t-tests: left hemisphere: t[39] = 2.20, p = 0.034; right hemisphere: t[39] = 2.41, p = 0.021). Demographic (age, education, IQ, handedness), behavioral (sleepiness, response time and hit rate for visual task), and MEG (the number of accepted sweeps for deviant stimuli, MMF peak latency) variables did not significantly differ between the genotypes (p's>0.32).

Variations in the other 3 SNPs did not significantly influence individual differences in MMF (main effect of genotype: p's>0.17).

## Discussion

Variations in *GRM3* rs274622 genotype are associated with individual differences in phonetic MMF power in the bilateral auditory cortices. To our knowledge, this is the first report that demonstrates the association between variations in a glutamate-related gene and individual differences in MMN/MMF in humans. These results confirm that MMF generation is regulated through glutamatergic neurotransmission and suggest that MMN/MMF may be a useful intermediate phenotype for genes coding the glutamatergic system in humans and possibly in patients with schizophrenia. In patients with schizophrenia, progressive decrease of gray matter volume of the superior temporal gyrus and MMN could concurrently occur [21], [22]. Therefore, the association between intermediate phenotypes and genes could be obscured to some extent in patients with schizophrenia. In this sense, MMF could be considered as both trait and state markers in schizophrenia. In contrast, young healthy subjects do not exhibit markedly progressive decline in these indices. Thus, MMF could be a relatively robust trait marker and the significant association between gene and intermediate phenotype could be predicted more clearly.

Bishop et al. [14] found that improvement in negative symptoms when treated with olanzapine was significantly greater in patients with schizophrenia who carry C allele of GRM3 rs274622 than in TT subjects. Taken together with the present findings, this SNP may be functionally relevant for cortical synaptic plasticity.

Our research group has previously demonstrated that effect size of the difference between healthy subjects and schizophrenia is larger for phonetic MMN/MMF than for tonal MMN/MMF [8], [9]. Thus, phonetic MMN/MMF may be a particularly sensitive intermediate phenotype for clarifying the molecular pathway of the glutamate system in the pathophysiology of schizophrenia.

Limitations of this study include small sample size and limited number of genes and SNPs investigated, and data on patients with schizophrenia being unavailable. Our next step should be a multivariate analytic approach to understand the complex relationship between various types of MMN/MMF and genes related to the glutamatergic system and synaptic plasticity identified through a genome-wide search in the general population and its alteration in patients with schizophrenia.
